# Multiple Carboxylase Deficiency in an Infant Presenting With Severe Metabolic Acidosis and Sepsis‐Like Features: A Case Report and Literature Review

**DOI:** 10.1002/ccr3.72990

**Published:** 2026-06-23

**Authors:** Touqeer Rehman, Riyan Saeed, Muhammad Ahmed Akif Rauf, Luqman Asif, Muhammad Junaid, Muddassir Khalid, Rao Nouman Ali, Umair Ali, Fred Segawa

**Affiliations:** ^1^ Ayub Medical College Abbottabad Pakistan; ^2^ Nowshera Medical College Nowshera Pakistan; ^3^ Abbottabad International Medical College Abbottabad Pakistan; ^4^ Medical Officer, Qazi Hussain Ahmed Medical Complex Nowshera Pakistan; ^5^ Khyber Medical College Peshawar Pakistan; ^6^ Department of Medicine Nishtar Medical University Multan Pakistan; ^7^ Consultant Urologist, District Headquarter Hospital Khanewal Pakistan; ^8^ Department of Pharmacy University of Swabi Swabi Pakistan; ^9^ Department of Medicine Makerere University College of Health Sciences Kampala Uganda

**Keywords:** biotinidase enzyme dysfunction of deficiency, case report, infant, metabolic acidosis, metabolic disorder, multiple carboxylase deficiency

## Abstract

Multiple carboxylase deficiency (MCD) is a rare, treatable inborn error of biotin metabolism that may present in children in the first year of life with life‐threatening metabolic crises. We report a 4‐month‐old child presenting with persistent seizures, eczematous rash near the orifices, unjustified loss of hair with baldness, and severe metabolic acidosis, initially mimicking septicemia. Urine organic acid investigation showed elevated levels of 3‐hydroxyisovaleric acid, 3‐methylcrotonylglycine, 3‐hydroxypropionic acid, methylcitrate, and lactate that lead towards MCD. This diagnosis remains unconfirmed, as confirmatory testing (serum biotinidase activity and genetic analysis) was not available at our center. Early initiation of biotin (10 mg/day) led to rapid clinical and biochemical recovery. This particular case depicts the importance of biotin‐responsive metabolic disorders in the first year of a child presenting with the classic triad of seizures, dermatitis, and alopecia with metabolic acidosis. Early recognition and prompt initiation of biotin therapy can result in rapid clinical recovery. Timely diagnosis is crucial to prevent avoidable morbidity and long‐term neurological sequelae. This case highlights the importance of considering MCD in children. In the first year of life, unexplained metabolic acidosis as timely treatment can be lifesaving.

**Abbreviations**
ABG
: Arterial Blood Gas
BTD
: Biotinidase Deficiency
CBC
: Complete Blood Count
CRP
: C‐Reactive Protein
CSF
: Cerebrospinal Fluid
GC–MS
: Gas Chromatography–Mass Spectrometry
HLCS
: Holocarboxylase Synthetase
IEM
: Inborn Error of Metabolism
LFTs
: Liver Function Tests
MCD
: Multiple Carboxylase Deficiency
RBS
: Random Blood Sugar
RFTs
: Renal Function Tests

**Key Clinical Message**

Multiple carboxylase deficiency should be considered in children in their first year of life presenting with unexplained metabolic acidosis and sepsis‐like features, especially when the child presents with a combination of seizures, alopecia, and dermatitis at the same time. Early recognition and prompt initiation of biotin therapy can result in rapid clinical recovery.

## 1 Introduction

Multiple carboxylase deficiency (MCD) is an autosomal recessive incompetent metabolism characterized by compromised activity of biotin‐dependent carboxylases. This deficiency may be caused by dysfunction of holocarboxylase synthetase (HLCS), which leads to the catalysis of covalent attachment of biotin to apocarboxylases, or biotinidase (BTD), which regenerates biotin from biocytin and dietary protein‐bound biotin [1]. As a result, accumulation of toxic organic acids occurs, showing clinically as dermatitis, seizures, and alopecia, in addition to high anion gap metabolic acidosis and hyperammonemia [2, 3]. The occurrence varies by region, with an overall prevalence of approximately 1 in 60,000 live births, between 1 in 40,000 and 1 in 126,000 in various populations [4].

Despite its rigorousness, MCD is still a treatable condition. Lifelong supplementation of biotin can reverse symptoms and prevent neurological complications if treated early [5, 6]. Although diagnosis is normally delayed due to the coinciding clinical features with other common early age conditions, such as meningitis, sepsis, or other metabolic disturbances [7]. Mortality rate is up to 70% in the untreated or late diagnosed cases [8, 9]. In patients with a delayed diagnosis, irreversible neurological dysfunction has been stated in 60 to 75% of patients [10]. These data don't clearly show the importance of maintaining clinical vigilance, particularly in a proven positive family history of child death. In this report, we illustrate a 4‐month‐old infant who presented with acidotic breathing, recurrent seizures, eczematous rash around orifices, and alopecia, with results consistent with MCD. This report underscores the clinical alertness required to differentiate this manageable disorder, focusing on the crucial role of metabolic assessments—including anion gap assessment, ammonia level, and urine organic acid analysis—as well as the promising therapeutic response to biotin, emphasizing the importance of early recognition in preventing irreversible complications.

This case report is reported in line with the CARE checklist.

## 2 Case History

**FIGURE 1:**
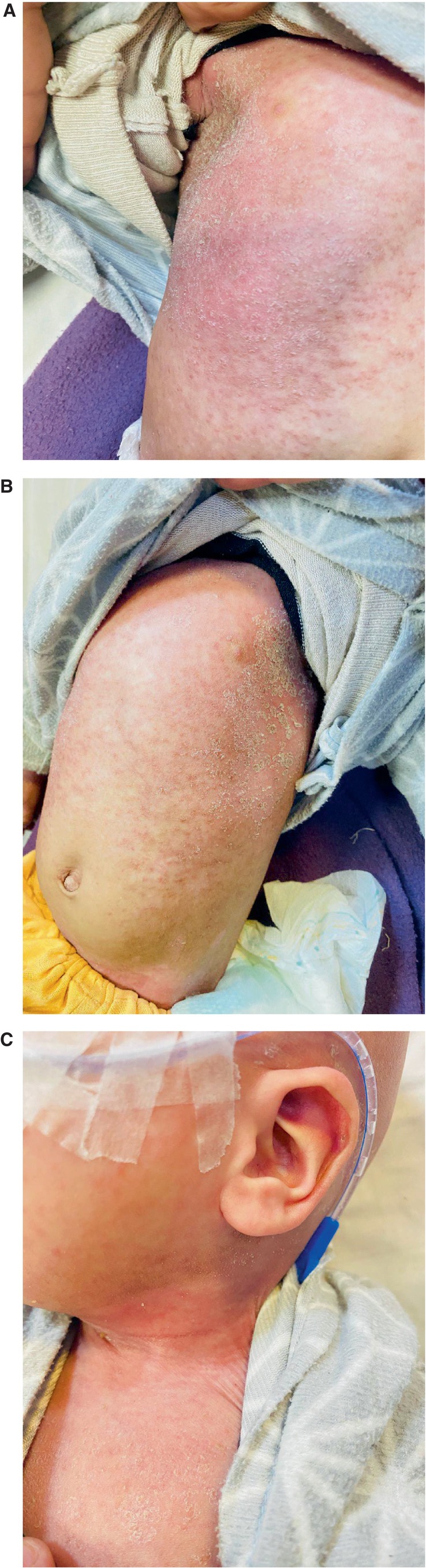
(A) Periorificial and axillary eczematous rash extending to the chest and abdomen. (B) Eczematous rash on the thorax and abdomen of the infant. (C) Eczematous rash involving the neck region.

**FIGURE 2:**
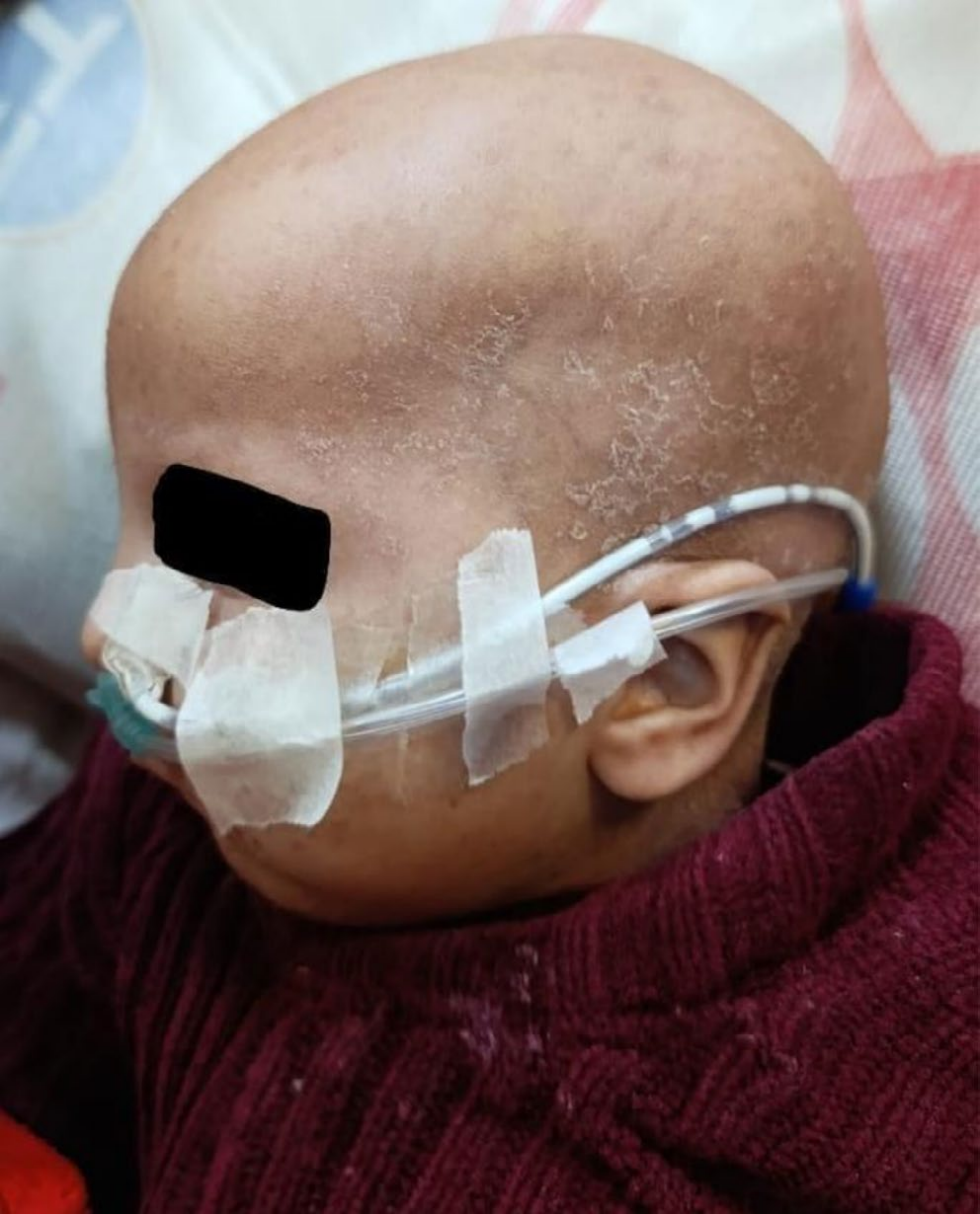
Sparse hair (alopecia) in a 4‐month‐old infant with suspected multiple carboxylase deficiency.

A 4‐month‐old male came to the pediatric Department of QHAMC (Qazi Hussain Ahmed Medical Complex Hospital, Nowshera, Pakistan) with unprovoked recurrent fits, acidotic breathing, a non‐healing red, scaly, eczematous rash near orifices, and sparse hair‐alopecia. Perinatal history was unremarkable. His parents have a consanguineous marriage, and he has one deceased sibling at two years of age with a history of seizures since birth. No such related case was seen in the extended family. On general examination, the patient was irritable and febrile (100.2°F / 37.9°C), and his skin showed periorificial and flexural rash [Figure 1A,B,C], and his hair was sparse [Figure 2]. Neurological workup revealed no signs of meningeal irritation despite seizures. The weight of the patient was 5.2 kg (< 3rd percentile), length was 60 cm (10th percentile), and OFC (occipito‐frontal circumference) was 40 cm.

## 3 Differential Diagnosis

Differential diagnosis can be grouped as follows [Table 1]:

**TABLE 1:** Differential diagnosis.

Category	Conditions
**Infectious Causes**	Secondary to lactic Acidosis could be viral or Bacterial Meningitis/Encephalitis
**Endocrine and Metabolic Disorders**	Propionic Acidemia, Methylmalonic Acidemia. Urea cycle disorders—Hyperammonemia with Encephalopathy Fatty Acid Oxidation Disorders Mitochondrial Disorders Congenital disorders—Galactosemia Congenital Adrenal Hyperplasia Vitamin B7‐related deficiency—Biotinidase deficiency
**Others**	Severe dehydration or shock, Perinatal Asphyxia, or Hypoxic ischemic Encephalopathy

Most likely could be Biotin‐Responsive metabolic disorder “Biotinidase Deficiency”,

- Classic presentation of triad (seizures, dermatitis, alopecia),
- Related to sibling death,
- Metabolic acidosis (high anion gap),
- Increased urine organic acids (3‐hydroxyisovaleric acid, 3‐methylcrotonylglycine)
- Infantile onset of symptoms (age of 4 months),
- Hyperammonemia.
- The features of skin relate more to the biotinidase deficiency than to the holocarboxylase deficiency.

## 4 Investigations

Results of investigations have been tabulated under Table 2:

**TABLE 2:** Investigations.

Category	Parameter	Result	Reference Range	Interpretation
**Hematology**	Hemoglobin	10.2 g/dL	~12–16 g/dL	Mild Anemia
	WBC count	11,500/μL	4,000–11,000/μL	Mild Leukocytosis
	Platelets	Normal	150,000‐450,000/μL	Normal
**Inflammation**	C‐ Reactive protein	8.2 mg/L	< 5 mg/L	Elevated
**Biochemistry**	Serum Calcium	10.1 mg/dL	8.6–10.2 mg/dL	Normal
	Random blood Glucose	95 mg/dL	70–100 mg/dL	Normal
**Liver Function Tests**	AST	56 U/L	10–4 U/L	Elevated
	ALT	48 U/L	7–56 U/L	Normal
	Total Bilirubin	0.7 mg/dL	0.1–1.2 mg/dL	Normal
	Albumin	3.8 g/dL	3.5–5.0 g/dL	Normal
**Renal Function**		Within normal limits		Normal
**ABG**	*p*H	7.18	7.35–7.45	Acidemia
	*p*CO2	22 mmHg	35–45 mmHg	Low (compensation)
	HCO3—	10 mEq/L	22–26 mEq/L	Low
	Base excess	–14 mEq/L	–2 to +2	Severe deficit
	Anion Gap	22 mEq/L	< 12 mEq/L	High anion gap metabolic acidosis
**Metabolic**	Serum Ammonia	207 μmol/L	< 50 μmol/L	Hyperammonemia
	Urine ketones	Positive	Negative	Ketosis
**Urine Organic Acids**	3‐hydroxyisovaleric acid, 3‐methylcrotonylglycine	Elevated		Suggests 3‐MCC deficiency
	3‐hydroxypropionic acid, methycitrate	Elevated		Suggests propionic acidemia
	Lactate	Elevated		Suggests pyruvate carboxylase dysfunction
**Overall metabolic pattern**				Multiple carboxylase deficiency (biotinidase vs. holocarboxylase)
**Imaging**	Abdominal Ultrasound	Normal		No abnormality
	Brain MRI	Normal		No structural/metabolic activity
	MR Spectroscopy	Not performed		
**Limitations**	Biotinidase assay/Genetic testing	Not done		Diagnostic confirmation Unavailable

## 5 Management

A lumbar puncture was performed to rule out meningitis. Results showed normal cerebrospinal fluid findings (cell count, protein, and glucose within normal limits). Cefotaxime 200 mg/kg/day in divided doses, Ampicillin plus Cloxacillin, and Levetiracetam were started empirically. Antibiotic therapy did not show any clinical improvement in the patient; later, a metabolic workup was done, including serum ammonia, arterial blood gas analysis, and urine organic salt analysis.

Results suggested multiple Carboxylase deficiency, Biotin was started with a dose of 10 mg/day orally. Seizures stopped within a week with biotin treatment. Along with eczematous rash, it started getting better in the first week of use of biotin and showed visible resolution within 4–6 weeks. Empirical therapy of antibiotics was stopped as the infectious etiology was excluded. Antiepileptic drug Levetiracetam was continued initially but tapered and stopped as the patient remained seizure‐free for 6 months on the use of biotin. Life long supplement of Biotin was advised to the patient.

## 6 Observation and Follow‐Up

The patients were observed every month in the first year, every 2 months in the second year, every 3 months in the third year, and every 6 months after 3 years. The cutaneous features had resolved completely in 4–6 months of biotin therapy and were still unremarkable. There was satisfactory hair growth by 3 months, and the patient achieved full growth. Hair density in 6 months. Other developmental milestones were achieved at the proper age as a healthy child. By 7 months of age, the patient sat independently, walked by 13 months of age, and spoke the first words by 12 months of age.

By 6 years of age, the patient is seizure‐free, has normal weight and height, satisfactory cognitive function, and has a healthy school routine. No evidence of recurrence of alopecia. Hearing and sight have been normal. Hence, the patient is advised to continue oral biotin 10 mg/day lifelong and should seek medical review if any symptoms occur.

## 7 Discussion

Multiple carboxylase deficiency is a rare metabolic disorder affecting approximately 1 in 60,000 individuals worldwide [11]. According to Baumgartner et al., multiple carboxylase deficiency, caused by impaired biotin metabolism, typically presents with neurological manifestations including hypotonia and seizures, and cutaneous findings such as alopecia [12]. Similarly, our patient presented with recurrent convulsions and eczematous rash with sparse hair (alopecia).

Molecular studies have shown that the enzymatic defect in multiple carboxylase deficiency differs depending on the age of onset. Early infantile‐onset disease (typically within the first few weeks of life) occurs due to holocarboxylase synthetase deficiency, although later infantile‐onset disease is due to biotinidase deficiency. In our patient, the symptoms that were evident at 4 months of age are more consistent with biotinidase deficiency [12, 13]. Studies have shown that patients with early age onset biotinidase deficiency often present with cutaneous manifestations, including patchy alopecia and skin lesions similar to acrodermatitis enteropathica [13]. Our patient exhibited sparse hair (alopecia) and a red, scaly eczematous rash, which relates to previously reported cutaneous findings.

Infants with multiple carboxylase deficiency also exhibit metabolic derangements, including hyperammonemia, metabolic acidosis, and organic aciduria [14]. Our patient presented with acidotic breathing, increased anion gap metabolic acidosis (high anion gap 22 mEq/L), high urinary organic acids, and hyperammonemia (207 μmol/L), all of which are very well related to metabolic derangements classically showing Multiple carboxylase deficiency. Some authors have also reported metabolic abnormalities in the cerebrospinal fluid, such as elevated alanine and lactate levels in patients with biotinidase deficiency [15]. In our patient, a lumbar puncture was performed to rule out meningitis. Lumbar puncture showed normal levels of cell count, protein, and glucose, but levels of lactate and alanine in cerebrospinal fluid were not analyzed specifically.

Biotinidase deficiency is readily treatable, and infant screening allows early diagnosis, which can prevent severe complications [8].

In this patient, after excluding infectious causes, an oral dose of Biotin was started at 10 mg/day. This therapy resolved cutaneous features within 4–6 weeks, and the patient became seizure‐free soon after the initiation of Biotin. Hair growth started to get normal by 3 months. At the age of 6 years, the patient had normal growth and an age‐appropriate response. The patient was advised to adhere to lifelong biotin supplementation to prevent recurrence.

Our center doesn't offer the facility to perform the confirmatory tests for the genetic analysis of the Biotinidase and Holocarboxylase genes, including specifically Serum Biotinidase activity assay and molecular genetic testing. Due to the patient's dramatic therapeutic response to the biotin therapy, the diagnosis remains presumptive and clinically based. The biochemical findings and clinical triad of the symptoms strongly favor the diagnosis. Yet, we cannot categorically exclude the Holocarboxylase synthetase deficiency without the confirmatory tests.

The case highlights the typical clinical symptoms of impaired biotin metabolic disorder and emphasizes the importance of early diagnosis and treatment to prevent undesired results. For a better understanding of the subject, we have also added the literature review of other reported early age onset cases in the last 5 years globally [Table 3].

**TABLE 3:** Literature review of infantile‐onset cases of multiple carboxylase deficiency reported in the last 5 years.

Author (year)	Journal (full name)	Diagnosis	Age/sex	Key Clinical presentation and course	Mutation/Enzyme status
Li Ke‐Yao et al. (2023)	Chinese Journal of Contemporary Pediatrics	HCD	16 mo/Male	History: Perioral erythema since the neonatal period; treated as dermatitis for 15 months. Acute: Aggravation of erythema, metabolic acidosis.	HLCS Gene Homozygous: c.1522C > T (*p*.R508W)
Kim et al. (2024)	Molecular Genetics & Genomic Medicine	HCD	8 days/Female	Acute: Severe lactic acidosis requiring mechanical ventilation; generalized ichthyosis. Outcome: Weaned off the ventilator 6 days after Biotin start.	HLCS Gene Compound Heterozygous: c.710 T > C; c.1544G>A
Gaschignard et al. (2024)	Frontiers in Genetics	HCD (Late Onset)	11 y/Female	Sister: Acute metabolic acidosis at age 11	HLCS Gene
			23 y/Male	Brother: Asymptomatic (diagnosed via family screening).	Homozygous: c.995A > G (*p*.Gln332Arg)
Zheng et al. (2025)	Orphanet Journal of Rare Diseases	HCD	Variable (4 Pts)	Biochem: Elevated 3‐hydroxyisovaleryl carnitine (C5OH). Note: Identification of novel variants in the Chinese cohort.	HLCS Gene Novel Missense: c.1505A>G Frameshift: c.2159delT
Nelson et al. (2024)	Clinica Chimica Acta	HCD	Infant/Female	Screening: Elevated C5‐OH on Newborn Screen (NBS). Confirmatory: Elevated urine 3‐hydroxyisovaleric acid.	HLCS Gene Homozygous Mutation (specific variant not listed in abstract)
Chetan et al. (2024)	BMJ Case Reports	BD	Neonate/*N*/A	Masquerade: Presented as Primary Immunodeficiency (septic arthritis, multiple abscesses). Neuro: Seizures.	Severe Biotinidase Deficiency (Enzymatic confirmation)
Kannan et al. (2024)	Molecular Biology Reports	BD (Profound)	10 mo/Male	Neuro: Seizures, hypotonia, ataxia, visual impairment. Biochem: Undetectable enzyme activity.	BTD Gene Biallelic Loss‐of‐Function: c.903G>A; c.946C>T
Singh et al. (2021)	BMJ Case Reports	BD	5 mo/Male	Neuro: Seizures, ataxia, developmental delay since birth. MRI: Diffusion restriction in the splenium of the corpus callosum & internal capsule.	Partial Biotinidase Deficiency Serum level: 0.2 nmole/min/mL
Cicalini et al. (2021)	International Journal of Environmental Research and Public Health	BD (Partial)	Newborn/Female	Metabolic: Altered acylcarnitine profile despite only “partial” deficiency. Detection: Identified via Newborn Screening.	BTD Gene Partial Variant (confirmed molecularly)

## 8 Limitations

This case has several limitations. First, the diagnosis remains presumptive; serum biotinidase activity assay and genetic testing (BTD and HLCS genes) were not performed due to unavailability at our center. These tests are essential to distinguish between biotinidase deficiency and holocarboxylase synthetase deficiency definitively. Second, MR spectroscopy, which could have provided additional metabolic information (e.g., elevated lactate in the brain), was not performed. Third, CSF lactate and alanine levels were not measured, which could have provided further biochemical evidence. We recommend that future cases be investigated with confirmatory enzymatic and genetic testing wherever possible.

## 9 Conclusion

This case highlights multiple carboxylase deficiency as a rare but manageable cause of early onset of metabolic crisis. Early recognition and prompt initiation of biotin therapy resulted in rapid clinical recovery, emphasizing the need to consider this diagnosis in children in the first year of life with unexplained metabolic acidosis, especially when dealing with alopecia, seizures, and dermatitis, to prevent morbidity and long‐term complications. Definitive diagnoses can only be established after serum biotinidase activity assay and genetic analysis.

## Author Contributions

**Touqeer Rehman:** conceptualization, formal analysis, writing – original draft. **Riyan Saeed:** methodology, writing – original draft. **Muhammad Ahmed Akif Rauf:** visualization, writing – original draft. **Luqman Asif:** visualization, writing – original draft. **Muhammad Junaid:** writing – original draft. **Muddassir Khalid:** project administration, supervision, writing – review and editing. **Rao Nouman Ali:** supervision. **Umair Ali:** writing – review and editing. **Fred Segawa:** writing – review and editing.

## Funding

The authors have nothing to report.

## Disclosure

The authors have nothing to report.

## Ethics Statement

Ethical approval is not required for case reports at our institution.

## Consent

Written informed consent was obtained from the patient's parents for publication of this case report and accompanying images. A copy of the written consent is available for review by the Editor‐in‐Chief of this journal on request.

## Conflicts of Interest

The authors declare no conflicts of interest.

## Data Availability

Data can be available on reasonable request from corresponding author.
